# Comprehensive Assessment of High-Risk Plaques by Dual-Modal Imaging Catheter in Coronary Artery

**DOI:** 10.1016/j.jacbts.2021.10.005

**Published:** 2021-12-27

**Authors:** Sunwon Kim, Hyeong Soo Nam, Min Woo Lee, Hyun Jung Kim, Woo Jae Kang, Joon Woo Song, Jeongmoo Han, Dong Oh Kang, Wang-Yuhl Oh, Hongki Yoo, Jin Won Kim

**Affiliations:** a Multimodal Imaging and Theranostic Lab, Cardiovascular Center, Korea University Guro Hospital, Seoul, South Korea; b Department of Cardiology, Korea University Ansan Hospital, Ansan-si, South Korea; c Department of Mechanical Engineering, Korea Advanced Institute of Science and Technology, Daejeon, South Korea; d Department of Biomedical Engineering, Hanyang University, Seoul, South Korea; e KI for Health Science and Technology, Korea Advanced Institute of Science and Technology, Daejeon, South Korea

**Keywords:** fluorescence lifetime imaging, high-risk plaque, inflammation, lipid, machine learning, optical coherence tomography, ch, channel, FL, fluorescence lifetime, FLIm, fluorescence lifetime imaging, ICC, intraclass correlation coefficient, IR, intensity ratio, IVUS, intravascular ultrasound, MФ, macrophage, OCT, optical coherence tomography, RFC, random forest classifier, ROI, region of interest, SMC, smooth muscle cell, TCFA, thin-cap fibroatheroma, UV, ultraviolet

## Abstract

Coronary plaque destabilization involves alterations in microstructure and biochemical composition; however, no imaging approach allows such comprehensive characterization. Herein, the authors demonstrated a simultaneous microstructural and biochemical assessment of high-risk plaques in the coronary arteries in a beating heart using a fully integrated optical coherence tomography and fluorescence lifetime imaging (FLIm). It was found that plaque components such as lipids, macrophages, lipids+macrophages, and fibrotic tissues had unique fluorescence lifetime signatures that were distinguishable using multispectral FLIm. Because FLIm yielded massive biochemical readouts, the authors incorporated machine learning framework into FLIm, and ultimately, their approach enabled an automated, quantitative imaging of multiple key components relevant for plaque destabilization.

## Highlights

• Fluorescence lifetime imaging (FLIm) allows label-free biochemical visualization of atheromas; however, it remains unknown whether FLIm can characterize high-risk plaque features in coronary arteries in a beating heart. Also, implementation of a novel analytic methodology is required for multispectral FLIm because it yields massive biochemical readouts.
• This study first demonstrated a simultaneous structural and biochemical assessment of high-risk plaques in the beating swine coronary arteries using a fully integrated optical coherence tomography-FLIm and a 2.9-F low-profile dual-modal catheter.
• Biochemical components of atherosclerotic plaques, including lipids, macrophages, lipids+macrophages, and fibrotic tissues, had unique fluorescence lifetime signatures that were clearly distinguishable using multispectral FLIm.
• Machine learning framework was successfully integrated with multispectral FLIm and enabled an automated, quantitative imaging of multiple key components associated with plaque destabilization.

## Visual Abstract

**Figure:**
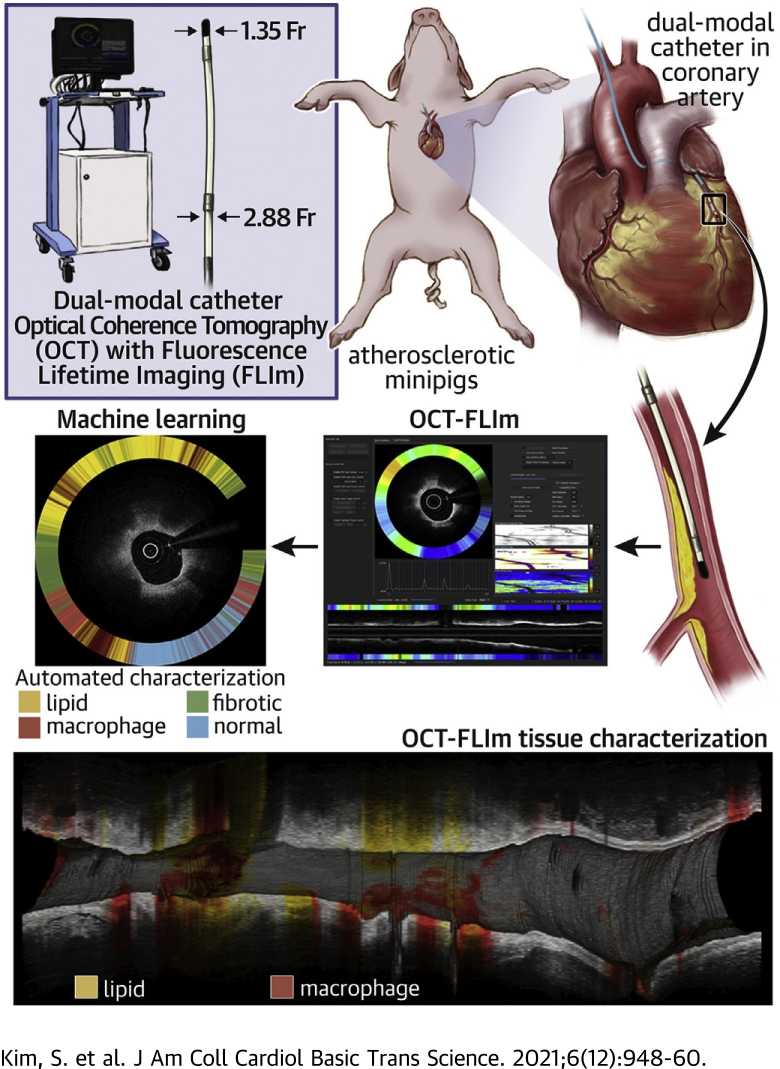

Coronary atherosclerosis and its thrombotic complication (ie, acute coronary syndrome) are leading causes of death worldwide (1). A lipid-rich, inflamed core and a thin overlying cap are proven as hallmark features of high-risk coronary plaques (2). However, given the highly complex multifactorial pathophysiology of atherosclerosis (3), conventional low-resolution anatomical imaging alone does not provide accurate detection of plaques at risk for future coronary events (4,5). Multimodal molecular imaging approaches enable detailed interrogation of plaque composition and molecular activity, and are thus expected to allow better risk assessment (6, 7, 8, 9, 10). However, the current multimodal molecular imaging method, albeit extensively studied and rapidly evolving, has limited clinical applicability because it inherently requires exogenous imaging agents with potential toxicity risks.

Fluorescence lifetime (FL) imaging (FLIm) is a novel approach allowing biochemical plaque characterization at molecular levels (11,12). FL, the time required by an excited molecule to return to the ground state via energy loss, is an intrinsic optical property of individual molecules (13). By utilizing FL as an endogenous contrast, FLIm allows detailed molecular component characterization without requiring exogenous imaging agents. Our group recently constructed a combined optical coherence tomography (OCT)-FLIm system and demonstrated that it could provide label-free structural-biochemical characterization of atherosclerotic plaques in vivo in rabbit arteries (14,15). Although atheroma development involves detectable alterations in the intrinsic fluorescence properties of the arterial wall (15,16), the ability of the fully integrated OCT-FLIm system to accurately characterize both the morphology and biochemical compositions of high-risk plaques has not yet been established in the coronary arteries of the beating heart. Furthermore, because advanced multispectral FLIm yields a massive amount of information (ie, channel-specific fluorescence intensity, intensity ratio [IR], and lifetime values), the immediate assessment of the key features in plaques is challenging (14).

Therefore, to resolve such issues, this study investigated: 1) whether our dual-modal intravascular OCT-FLIm system is able to characterize high-risk coronary plaque features in vivo in a coronary artery of the beating heart; and 2) whether a machine learning-based approach can be successfully integrated with multispectral OCT-FLIm for automated, real-time biochemical characterization to detect high-risk coronary atheromata.

## Methods

### Dual-modal OCT-FLIm system

We developed a dual-modal fiber-optic hybrid rotary joint and a 2.9-F highly flexible imaging catheter for in vivo intracoronary imaging of combined OCT-FLIm (Supplemental Figure 1) (14,15). This imaging system allowed rapid image acquisition with a maximum pullback speed of 40 mm/s and a rotational speed of up to 100 rotations/s (15). For multispectral FL measurements, returning tissue autofluorescence was temporally and spectrally resolved into 3 different spectral channels (ch) with a wavelength/bandwidth of 390/40 nm (ch.1), 452/45 nm (ch.2), and 542/50 nm (ch.3). The IR, a relative fluorescence intensity between different channels, was quantified to obtain distance-insensitive autofluorescence information. The present FLIm based on ultraviolet (UV) light had a penetration depth of around 200 μm and produced a total of 3,072 readouts for each frame (3 channel FLs and 3 IR values per every 4 OCT A-line locations). Each FL and IR was color-coded and displayed at the corresponding OCT A-line. In this paper, only ch.2 FL information is displayed for simplified visualization (ch.1 FL and IR data are provided in Supplemental Figures 2 to 4). Ch.3 data were not analyzed, because the channel’s fluorescence intensity has insufficient signal-to-noise ratio in the swine arteries (16). The radiation value was 0.46 mJ/cm^2^ on the arterial walls, which is much lower than the maximum permissible exposure value for the skin (3.52 mJ/cm^2^) according to the laser safety guidelines (*American National Standard for Safe Use of Lasers in Health Care*; ANSI Z136.3) (17,18).

### Animals, in vivo OCT-FLIm, and histologic validation

Four Yucatan minipigs (sex male, age 3 to 5 months, weight 15 to 20 kg; Optipharm) were used in this study. Three high-fat-diet–fed, diabetic minipigs were subjected to balloon overstretch injury at the left anterior descending artery to induce accelerated coronary atherosclerosis, as previously described (Supplemental Figure 5) (10). One regular-chow–fed minipig served as a normal artery model. After imaging, the coronary arteries were serially sectioned and immunostained to identify lipids (Oil Red O), macrophages (MФs; PM-2K), and smooth muscle cells (SMC; smooth muscle actin [SMA]). The Institutional Animal Care and Use Committee of Korea University approved the study (KUIACUC-2017-0019 and KOREA-2018-0070) (see the Supplemental Appendix for more details).

### FLIm analyses according to different plaque compositions

For composition-specific FL analysis, the plaque areas were divided into regions enriched in fibrotic components, either lipids or MФs, or both (lipids+MФ), based on the immunohistology findings. A region of interest (ROI) was drawn for each component based on the OCT and corresponding immunohistology findings, and the multispectral FL measurements at every OCT A-line location included in the ROI were analyzed.

### Machine learning–based plaque component characterization

A random forest classifier (RFC) (19), a machine learning algorithm for multiclass classification, was harnessed for biochemical plaque characterization and trained to transform sets of multispectral FLIm data feature maps (ch.1 and ch.2 FLs and IR) into plaque component maps with 5 different classes: lipids, MФs, lipids+MФ, fibrotic, and normal wall. We trained the RFC with the sets of OCT-FLIm ROIs, which were determined on the basis of the histology as described in the preceding text. A total of 297 ROIs obtained from 3 different animals (2 atheromatous models and 1 normal artery model) were used for RFC training and cross-validation. The classification results were color-coded as follows: normal wall with bright blue; fibrotic tissue with green; lipids with yellow; MФs with bright red; and lipids+MФ with dark red.

### Reliability and reproducibility validations

We compared 2 repeated imaging datasets to evaluate whether the current FLIm reliably estimates FLs and whether the trained RFC classifies plaque components in a reproducible manner. Two imaging experts (S.K. and J.W.S.), blinded to the FLIm data, paired OCT frames based on the morphological characteristics, such as side branches, intimal neovessels, plaque shape, and plaque size. The pairing was accomplished with a high level of interobserver agreement (46 of 49 pairs). The RFC accuracy was further evaluated by comparing RFC-determined plaque components versus quantitation from immunohistochemistries. For this purpose, digitized immunohistochemistry images were color-decomposed to separate the stain-positive areas using a digital image analysis algorithm (20). Each image was then color-coded (lipid with yellow, MФ with red, and SMC with bright blue) and overlaid together to generate a multiplexed immunohistochemistry image. Considering the FLIm penetration depth, a 200-μm–depth quadrangular ROI was drawn at every 1-degree angle interval along the luminal surface. The densities of each component within the ROI were morphometrically quantified and compared with in vivo RFC results. The component with the highest relative density was regarded as the representative plaque component of each ROI.

### Statistical analysis

Values are expressed as mean ± SD. Differences in FL measurements were analyzed via 1-way analysis of variance using Tukey's post hoc method for pairwise comparisons or the Kruskal-Wallis test using Dunn's post hoc test based on the normality test results. The multispectral FL measurements were averaged for each frame (plaque-type–based FL analysis) or each ROI (component-specific FL analysis) before comparisons. Agreement of the multispectral FL measurements or RFC results across repeated imaging datasets was evaluated using Bland-Altman analysis and intraclass correlation coefficient (ICC) analysis. All analyses were conducted using Prism software version 8.0 (GraphPad Software,) and SPSS software version 20.0 (IBM SPSS Statistics). Volume-rendered images were generated using the DICOM viewer, OsiriX (The OsiriX Foundation).

## Results

### In vivo OCT-FLIm

**Figure 1:**
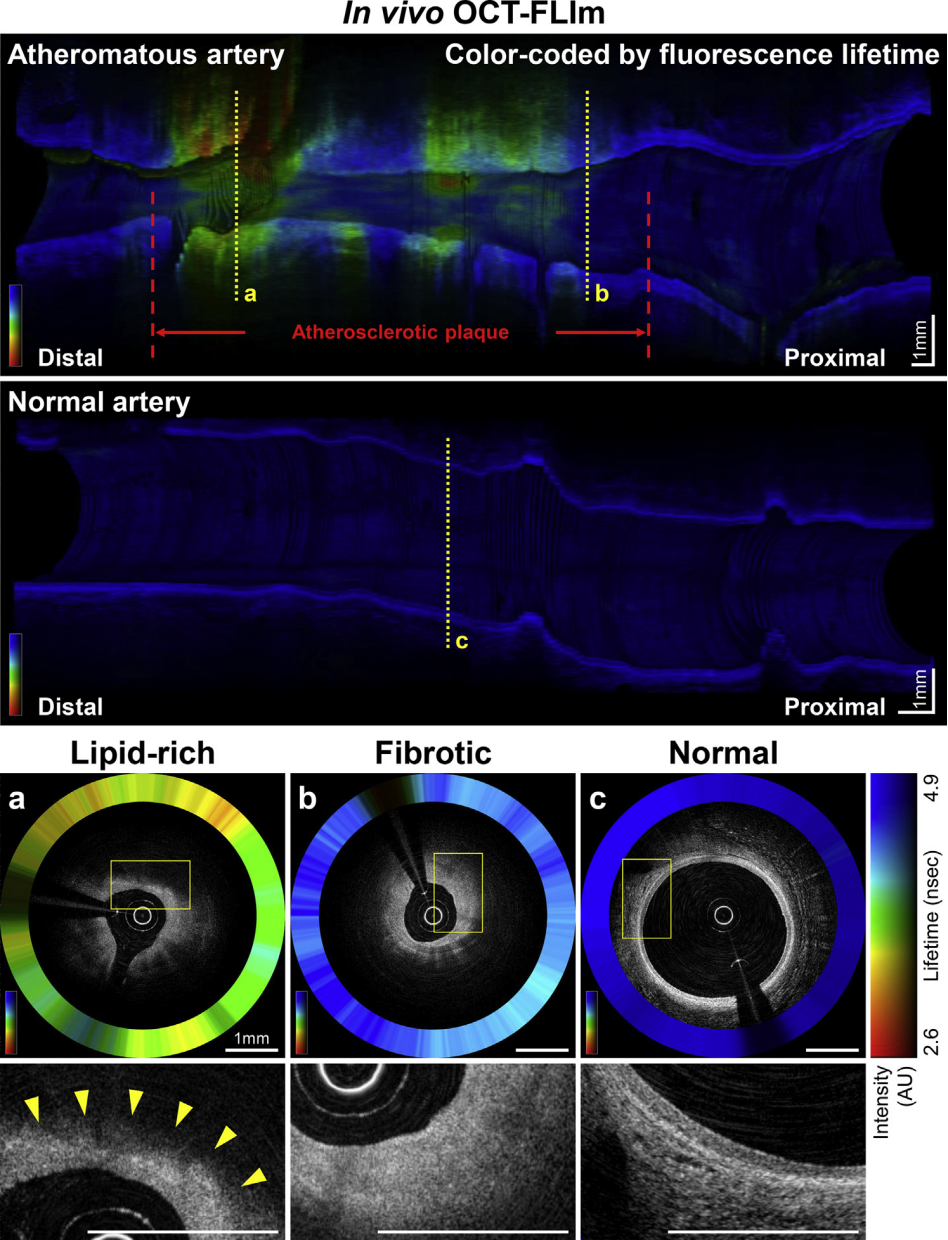
In Vivo OCT-FLIm Images Obtained From an Atheroma and a Normal Artery **(Upper panel**) Three-dimensional longitudinal cutaway-view images color-coded with the ch.2 FL. **(Lower panel**: **a, b, and c)** OCT-FLIm cross sections at the corresponding locations. **Boxedareas in a, b, and c** are shown in higher magnification in the panels **below**. **Arrowheads (a)** denote typical signal-poor OCT regions of lipid-rich plaques, whereas fibrotic plaque **(b)** shows homogenous, signal-rich OCT regions. Scale bars indicate 1 mm. AU = arbitrary units; ch = channel; FL = fluorescence lifetime; FLIm = fluorescence lifetime imaging; OCT = optical coherence tomography.

All rapid intracoronary imagings (pullback speed 10 to 20 mm/s) were safely performed in vivo under Dextran 40 solution purge. Along with high-resolution anatomical imaging via OCT, multispectral FLIm provided coregistered biochemical readouts from the target plaques (Figure 1, Videos 1 and 2). The dedicated data analysis software provided real-time display of coregistered OCT-FLIm images and reconstructed en face FL maps within seconds (Supplemental Figure 6, Video 3). After stratifying in vivo OCT-FLIm images based on the qualitative OCT characteristics (Figure 1) (21,22), significant differences in FL signatures were identified between lipid-rich plaque versus fibrotic plaque versus normal wall (*P* < 0.001) (Supplemental Figure 7) (see the Supplemental Appendix for more detailed methods and results).

### Component-specific FLIm analysis

**Figure 2:**
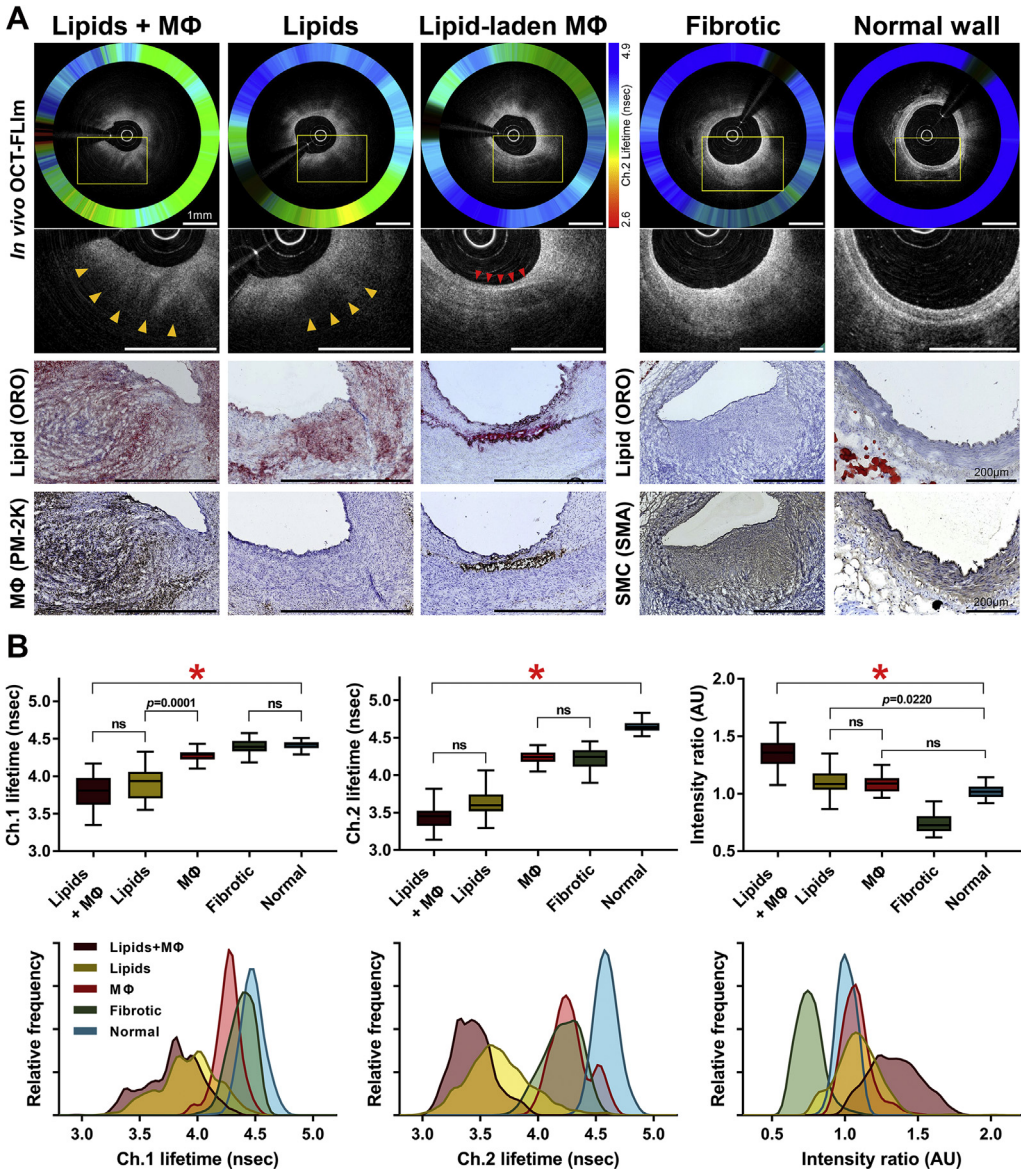
Component-Specific FLIm Analyses **(A)** FLIm signatures according to the different plaque components. Each column consists of original and higher-magnified OCT-FLIm images and corresponding immunohistologies. **Boxedareas in the top panels** are shown in higher magnification in the panels immediately **below**. Scale bars indicate 1 mm, unless otherwise specified. **Yellow arrowheads** denote typical signal-poor, lipid-rich regions on OCT. **Red arrowheads** denote typical OCT bright spots, suggesting macrophage accumulation. **(B)** Comparisons of multispectral FLIm measurements according to the plaque components (∗*P* < 0.001 by the Kruskal-Wallis test). Boxplot center lines indicate the medians; box edges represent the interquartile ranges; and whiskers extend to the first or third quartile plus 1.5 times the interquartile range. The multiple comparison results all yielded *P* < 0.001, unless otherwise specified or indicated as nonsignificant (ns). MФ = macrophage; ORO = Oil Red O stain; other abbreviations as in Figure 1.

We analyzed the FL values from the plaque ROIs, which were classified as 5 different types: fibrotic (n = 79), lipid-rich (n = 66), MФ-abundant (MФ, n = 41), lipid-rich with abundant MФs (lipids+MФ, n = 38), and normal artery (n = 73) (Figure 2A). The ch.2 FL histogram from MФs showed a bimodal distribution, wherein 1 peak represented lipid-laden MФs residing superficially at the vessel surface, and the other peak represented those clustered at the intima–media interface (Supplemental Figure 8). The mean ch.1 FLs of the compositional types differed significantly, except for the values between lipids+MФ-rich versus lipid-rich regions (*P* > 0.99) and between fibrotic regions versus normal wall (*P* > 0.99). The mean ch.2 FLs also differed significantly across each other, except for the values between lipids+MФ-rich versus lipid-rich regions (*P* = 0.72) and between MФ-rich versus fibrotic regions (*P* > 0.99) (Figure 2B). Although the lipids+MФ-rich and lipid-rich regions exhibited similar mean ch.1 and ch.2 FLs, there was a prominent difference in the IR (*P* < 0.001), implicating that the current multispectral FLIm could differentiate between them. Similarly, each biochemical component was distinguishable from one another by the differences in ch.1, ch.2 FLs, and IR. The mean ch.1 and ch.2 FLs and IR values as well as the multiple comparison results across the 5 different components are shown in Supplemental Tables 1 and 2.

### Machine learning–based automated, quantitative plaque compositional characterization

**Figure 3:**
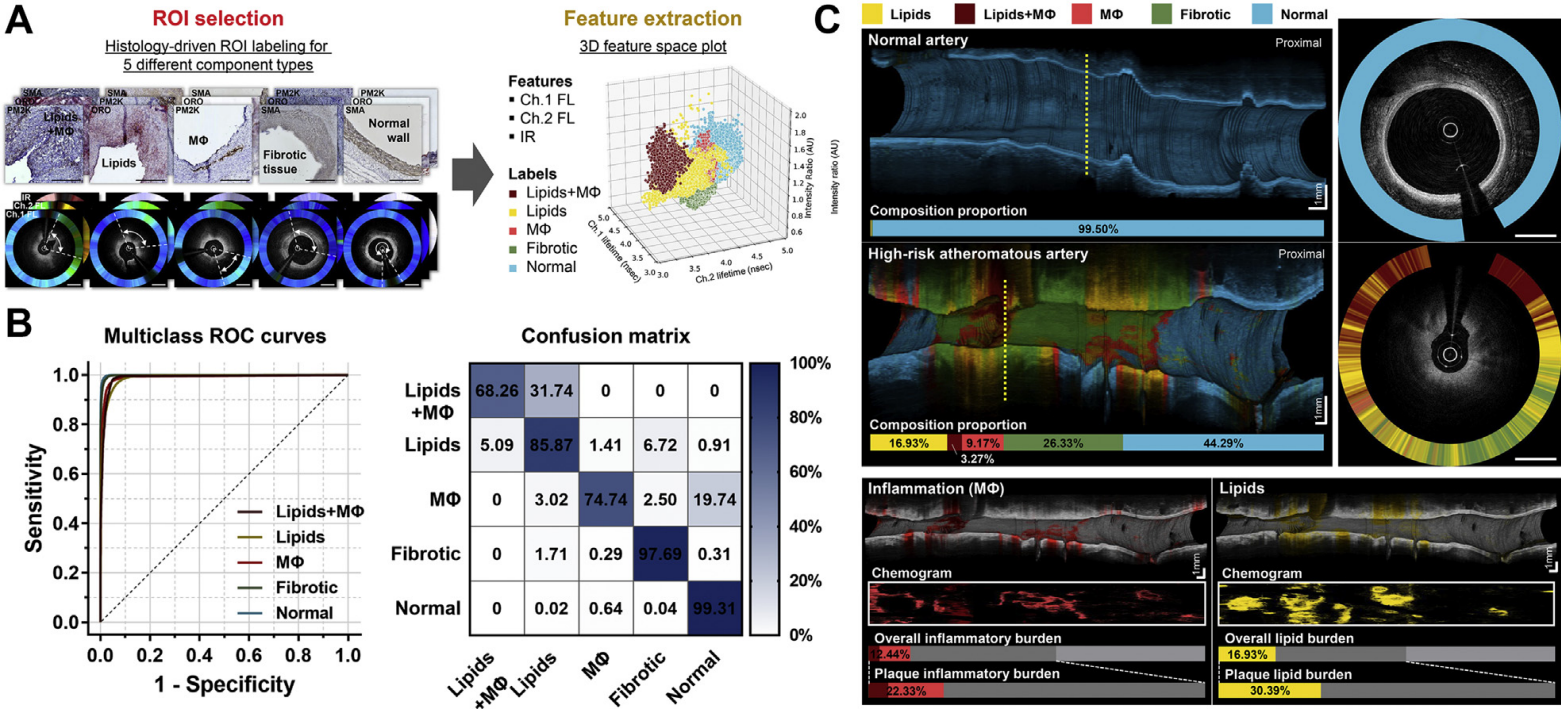
Machine Learning–Based Automated Biochemical Characterization **(A)** Dataset was assembled by obtaining biochemical FLIm readouts from the predetermined regions of interest (ROIs, **arrowheads**) labeled with the 5 different classes. In the training phase, each random forest classifier (RFC) decision tree was formulated using a randomly sampled training subset. **(B)** The trained classifier performance was evaluated using the confusion matrix and multiclass receiver operation characteristic curve analysis based on five-fold cross-validation. **(C)** Cross sections and volume-rendered images of the RFC-applied OCT-FLIm. This imaging approach enables intuitive visualization of the structural-biochemical characteristics of target plaques and quantitative composition analysis of the 5 different biochemical components. Abbreviations as in Figures 1 and 2.

We evaluated whether a machine learning classifier, trained with component-labeled multispectral FLIm datasets, could identify the key biochemical compositions of high-risk plaques accurately and automatically. A RFC algorithm was successfully applied, achieving a robust classification performance with an overall 5-fold cross-validation accuracy of 94.4% and with high sensitivity (97.0% to 99.1%) and specificity (92.8% to 98.4%) across the plaque classifications for the 5 components (Figures 3A and 3B, Supplemental Table 3). The RFC utilized the high-dimensional data integrating the ch.1 FL, ch.2 FL, and IR altogether and provided final classification results in real time (Video 3). The RFC-incorporated FLIm was able to provide biochemical plaque readouts on a frame-by-frame basis (Supplemental Figure 9, Video 4) and also allowed comprehensive compositional characterization of the entire scanned artery itself (Figure 3C, Video 5). Furthermore, it provided the quantitative compositional burden index of each biochemical component, which summarized the amount of each constituent in the entire scanned segment on a 0%-to-100% scale (Figure 3C). We further tested the algorithm in the swine that was excluded from the training and found that the RFC was able to consistently classify the plaque components with high accuracy (Video 6).

### Reliability and reproducibility analysis

**Figure 4:**
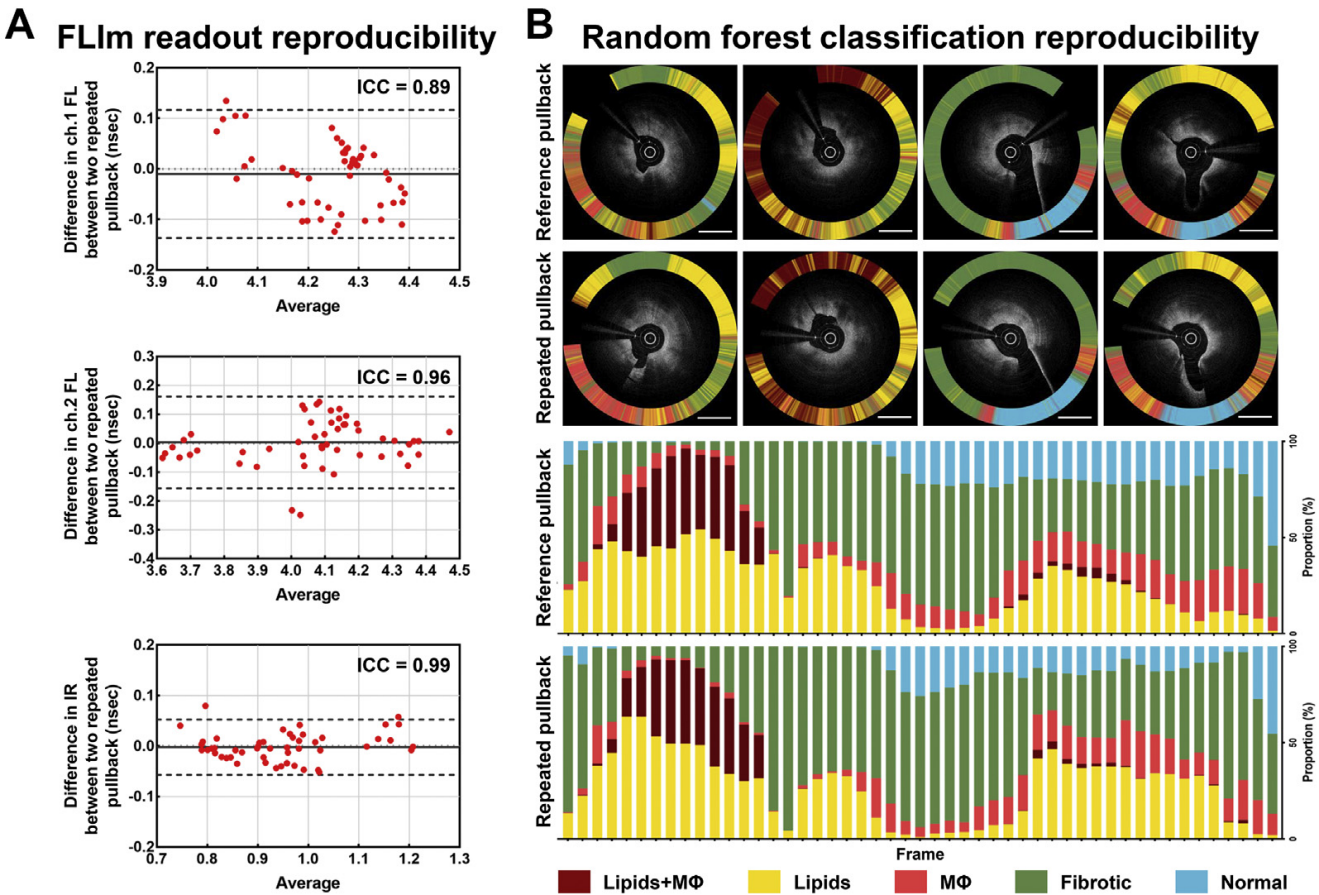
Reliability and Reproducibility Validations **(A)** Scatter and Bland–Altman plots showing correlations of the multispectral FLIm measurements between the repeated imaging datasets. Bland-Altman graph: The differences between the repeated imaging datasets were plotted against their mean, and the 95% limits of agreement **(dashed line)** were calculated as the average difference ± 1.96 SD. **(B) (Upper panel)** Representative images of the paired frames showing similar classification results. **(Lower panel)** Stacked bar graphs showing the relative proportions of the RFC-determined plaque components. Each **vertical bar** along the horizontal axis represents a frame. **Scale bars** indicate 1 mm. ICC = intraclass correlation coefficient; IR = intensity ratio; other abbreviations as in Figures 1 to 3.

To examine the reliability and reproducibility of the current FLIm approach, we compared 49 pairs of 187 plaque-containing frames from 2 repeated imaging datasets. The mean ch.1 and ch.2 FLs and IR showed high agreement across the repeated pullbacks (mean ch.1 FL: ICC = 0.90; *P* < 0.001; mean ch.2 FL: ICC = 0.96; *P* < 0.001; IR: ICC = 0.99; *P* < 0.001). In the Bland-Altman plots (Figure 4A), the data points were placed within the agreement limits, and no significant systematic biases were noted. The relative proportions of RFC-determined plaque components were highly consistent across the repeated pullbacks (lipids+MФ: ICC = 0.99; *P* < 0.001; lipids: ICC = 0.88; *P* < 0.001; MФ: ICC = 0.87; *P* < 0.001; fibrotic: ICC = 0.96; *P* < 0.001; normal wall: ICC = 0.94; *P* < 0.001) (Figure 4B).

### Machine learning–incorporated FLIm for inflammation imaging

**Figure 5:**
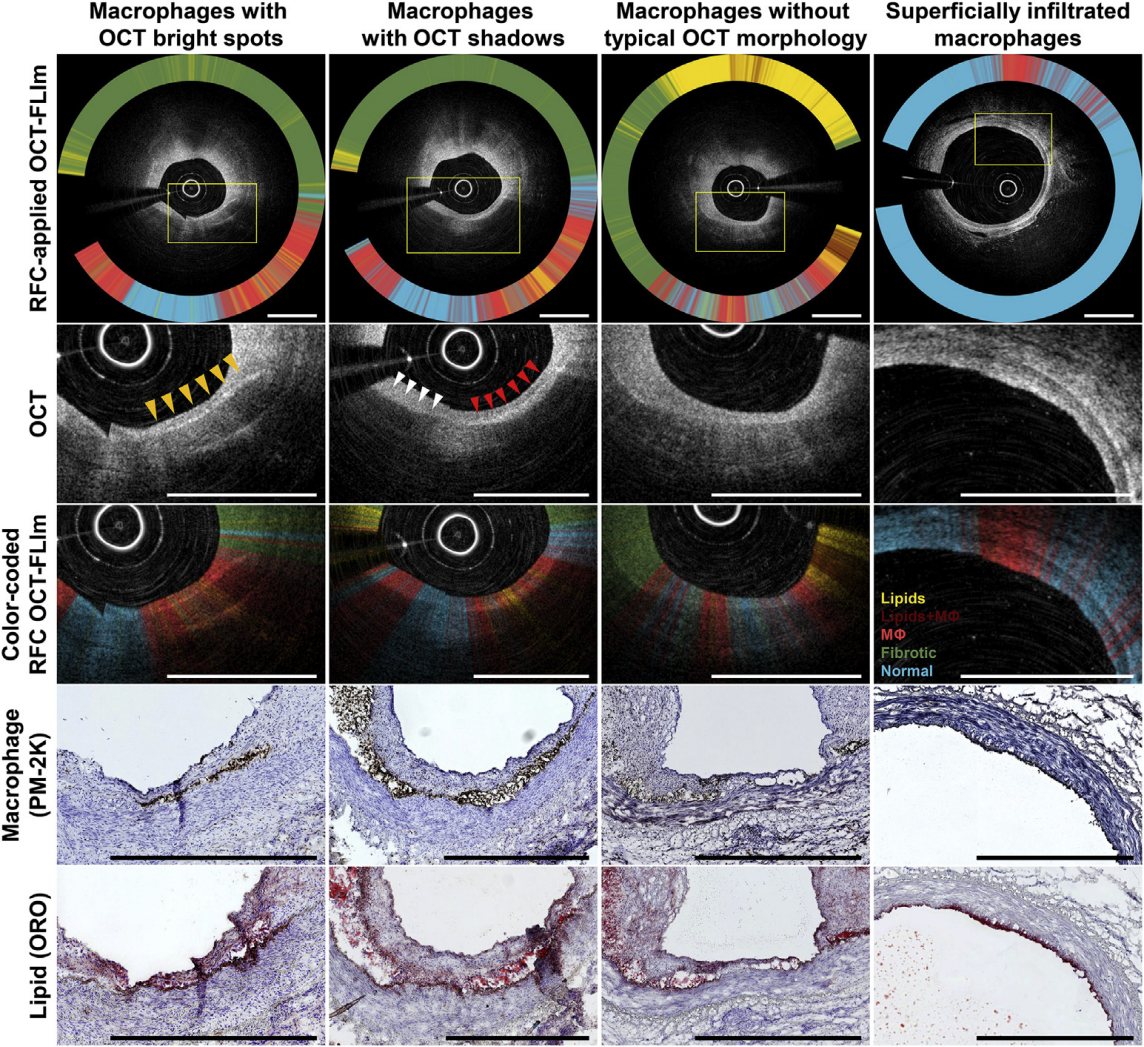
Macrophages Showing Various Morphological Features on OCT **Yellow arrowheads** indicate typical OCT bright spots without shadows; **white arrowheads** indicate only shadows in the absence of bright spots; and **red arrowheads** show typical OCT bright spots with shadowing. **Boxedareas in the top panels** are shown in higher magnification in the panels immediately **below**. The RFC-applied FLIm allowed highly sensitive and accurate detection of macrophage infiltrates. Scale bars indicate 1 mm. MФ = macrophage; other abbreviations as in Figures 1, 2, and 3.

Most OCT bright spots being generated by the lipid-laden MФs were densely clustered at the interface between the intima and media (23); however, the plaque MФs exhibited a highly variable morphology on OCT (eg, typical bright spots with or without shadowing, or only shadowing without bright spots) (Figure 5). Moreover, OCT alone could not effectively characterize focal or minimal MФ infiltrates, whereas our machine learning-incorporated FLIm was able to precisely localize the MФ accumulations regardless of their appearance on OCT (Figure 5).

### Ex vivo quantitative immunohistochemistry validation

**Figure 6:**
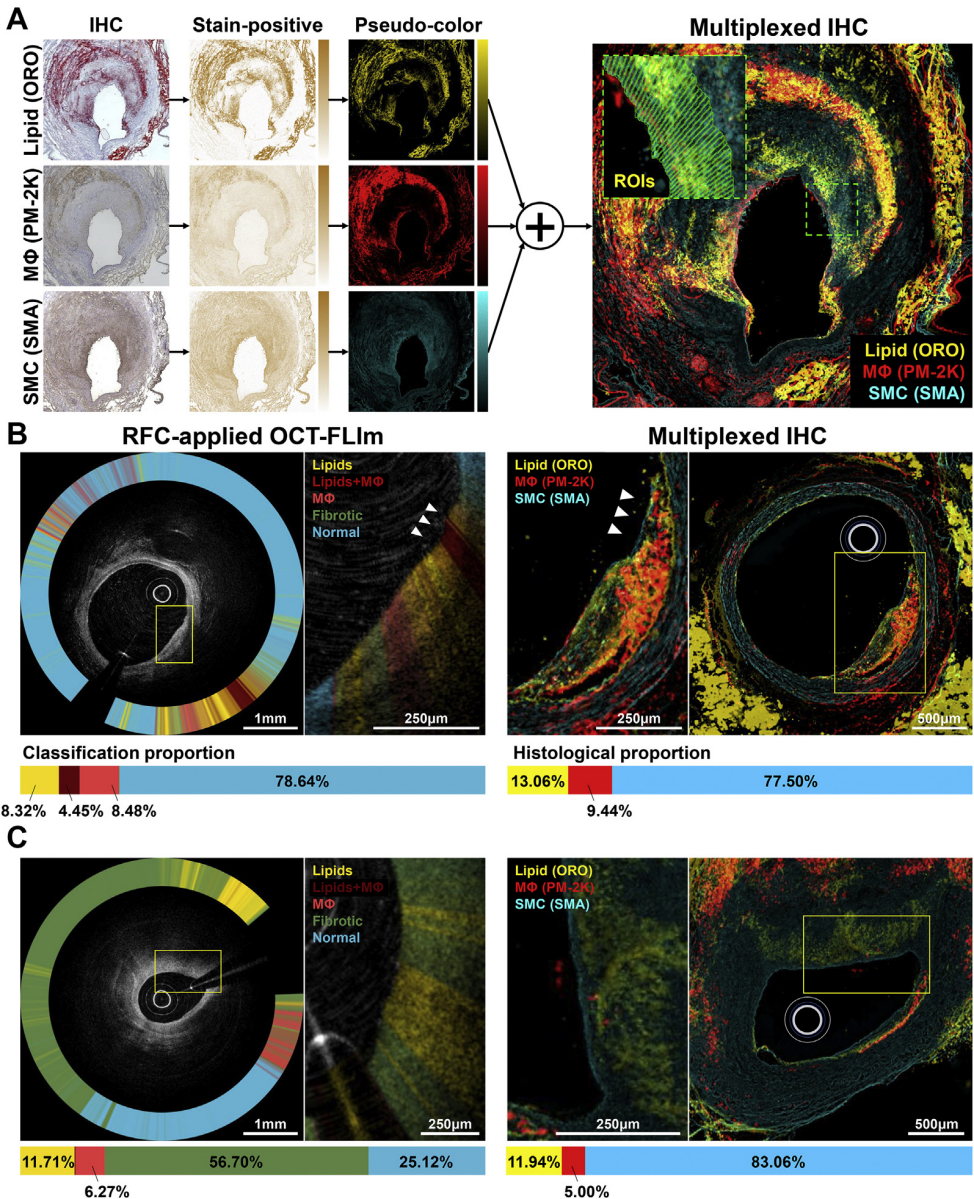
Comparison Between the RFC-Determined Plaque Components and Those Quantitated From Immunohistochemistries **(A)** Flowchart describing the process of the immunohistochemistry (IHC) multiplexing. **Inset** (a higher-magnification image of the **dashed-line boxed area**)**,** each **green rectangle** denotes the 200-μm–depth quadrangular ROI for morphometric quantitation. **(B)** Focal plaque showing extensive macrophage infiltration and thinning of the overlying SMC layer at the plaque shoulder (**arrowheads,** also refer to Supplemental Figure 10). **(C)** Comparison result of an atheroma section obtained from the animal whose imaging data were not used for RFC training. **Boxed areas** are displayed in high-magnification in neighboring images, accordingly. SMA = smooth muscle actin; SMC = smooth muscle cells; other abbreviations as in Figures 1, 2, and 3.

The trained RFC accurately identified high-risk features, such as the lipid-rich, highly inflamed region (lipids+MФ: dark red) at the plaque shoulder, leading to thinning of the overlying fibrotic layer (Figure 6B, Supplemental Figure 10). The RFC-determined in vivo OCT-FLIm composition corresponded well with the quantitated immunohistology findings (Figure 6B). In the plaque section obtained from the swine not utilized for RFC training, the RFC accurately detected lipid accumulations within a grossly fibrotic intima (at 12 to 1 o’clock) and MФ infiltrates (at 3 to 4 o’clock) (Figure 6C). Consistently, the RFC-determined biochemical compositions corresponded well with the quantitation from the multiplexed immunohistochemistries (Figure 6C).

## Discussion

In the present study, we constructed a fully integrated, high-speed OCT-FLIm system and a low-profile dual-modal catheter, which is able to provide both clinical-grade OCT plaque images and its coregistered compositional multispectral FLIm information in the beating coronary arteries. Through rigorous histological validations, we demonstrated that the key biochemical plaque components, such as lipids, MФs, and SMC-rich fibrous tissues, had unique, multispectrally distinguishable FL signatures, suggesting that our cutting-edge intravascular OCT-FLIm has the capability to simultaneously characterize them in vivo in a label-free manner. Because FLIm yields massive multispectral readouts that are not intuitively recognizable, we incorporated the machine learning algorithm to FLIm; ultimately, our OCT-FLIm could allow an automated and quantitative characterization of high-risk plaque components in a highly reproducible manner.

Atherosclerotic plaque progression involves intimal SMC recruitment in response to lipoprotein accumulation and related immune activation (3,24). SMCs produce collagen- and proteoglycan-rich extracellular matrices pivotal to plaque stabilization (25), whereas excessive, maladaptive inflammatory responses by MФs degrade the supporting framework, leading to cap thinning and rupture (26). Thus, the assessment of these key biochemical components offers quantitative measures for stratifying individual plaque risks. Indeed, recent studies using combined intravascular ultrasound (IVUS) and near-infrared spectroscopy have shown that the acquisition of additional biochemical information, albeit a standalone lipid chemogram, could not only enhance diagnostic accuracy in determining high-risk plaques (27), but also allow identification of patients and arterial segments at risk for developing future adverse events (28).

In addition to lipids, MФ is a key component that participates in every stage of atherosclerosis, from initiation to eventual plaque destabilization; thus, in vivo assessment of these cellular activities is critical for enhancing our understanding of plaque behavior and, more importantly, for preventing its lethal complications (3,26,29). However, there has been a lack of imaging modality that enables quantitative inflammation imaging of a small coronary plaque. Although previous studies have shown the feasibility of OCT in identifying MФs (30,31), its practicability remains controversial (23). Plaque MФs exhibited highly variable OCT features, hindering reliable detection and quantitation of plaque inflammation. The present FLIm provided not only a highly sensitive and reliable readout of MФ infiltrates, but also quantitative indices of key components, including MФs, lipids, and fibrosis. This OCT-FLIm enabling quantitative imaging of multiple biochemical characteristics in the context of plaque morphology will offer a more sophisticated measure for assessing plaque behavior and monitoring the efficacy of therapeutic modulation.

Among various pathological characteristics of coronary atherosclerotic plaques, fibrous cap thickness was found to have the highest hierarchical importance in determining plaque vulnerability (2). Although OCT is the sole imaging modality with sufficient resolution to characterize thin-cap fibroatheromas (TCFAs) (21), false identification of TCFAs using standalone OCT has been reported (32,33). Plaque components that scatter light to create false TCFA images varies widely: MФs, hemosiderin, fibrin, microcalcification, proteoglycan-rich loose connective tissue, and even densely packed SMCs themselves (32,33). FLIm yields quantitative biochemical readouts irrespective of light scattering or absorption (13) and thus should aid in accurate TCFA diagnosis. The utility of OCT in differentiating lipid pools with necrotic cores is limited because light attenuation is commonly seen in both lipid pools and MФ-rich necrotic lesions (22). FLIm allowed differentiation between plaques infiltrated with lipids only versus those enriched in both lipids and MФs, demonstrating its capability to further characterize plaque cores. FLIm identification of MФ clusters beneath the fibrotic layer also implies its potential for assessing cap inflammation.

To our knowledge, this is the first intracoronary demonstration of OCT-FLIm for detecting plaque components associated with high-risk human plaques, which is successfully integrated with a machine learning framework. A recent study using combined IVUS and FLIm reported in vivo imaging results obtained from healthy and stented swine coronary arteries (16); however, the clinical applicability of IVUS-FLIm remains controversial. Given the inherent limitation of FLIm requiring blood elimination, rapid image acquisition is essential to reduce the risk of myocardial ischemia. However, with the current IVUS technology, rapid imaging at sufficient frame rates to achieve reasonable luminal sampling is challenging. Although a novel high-frequency (>60 MHz) IVUS was reported to offer a more rapid image acquisition at 60 frames/s and a more rapid pullback speed of up to 10 mm/s (34), it is still below the level of contemporary OCT. Essentially, such combination undermines the foremost merit of IVUS, that is, the capability to visualize blood-filled vessels.

The present dual-modal imaging system has several additional advantages that can facilitate its translation into catheterization laboratories. The dual-modal imaging probe has favorable catheter profiles comparable to those of clinical OCT. This fluorescence imaging approach, exploiting FL as an endogenous contrast, only requires additional UV radiation at levels far below the maximum permissible exposure limit. Moreover, as the current FLIm system was established on the basis of a novel high-speed FL measurement methodology (14), its imaging speed, albeit already at a clinical level, can be extended by simply increasing the pulse repetition rate of the light source. Furthermore, as the dedicated analysis software offers rapid data processing and real-time presentation of multispectral imaging data including RFC results, we believe that the present OCT-FLIm system is ready for clinical translation.

The current FLIm based on UV light has a limited penetration depth of around 200 μm and lacks depth-resolved imaging capability. Fluorescence imaging techniques, such as confocal and multiphoton excitation microscopy, are known to provide depth-resolved images (35,36). However, time-consuming depth scanning may greatly reduce the image acquisition speed; thus, it is challenging to apply these techniques to OCT-based multimodal imaging, which acquires images via a rapidly rotating probe while eliminating the coronary blood flow. Nevertheless, key high-risk plaque features, including thin cap (<65 μm), lipid or necrotic core abutting to the lumen, cap inflammation, superficial calcium, and healed thrombus, are certainly within the limit of FLIm penetration (37,38). A recent study has shown the feasibility of simultaneously acquiring OCT, IVUS, and fluorescence data using a trimodality imaging probe (39). The integration of FLIm into a high-speed trimodality catheter approach will be promising, as it can assess both superficial and deeper plaque structures as well as multiple biochemical characteristics associated with high-risk plaques.

The RFC is an ensemble-supervised machine learning technique that combines a large number of decision trees and makes its final decision by obtaining the majority voting of individual classifiers (19). It shows robust performance with a low risk of overfitting even on noisy data (19), which is an unavoidable pitfall in medical imaging. Accurate and comprehensive training datasets are critical for the performance of any machine learning–based approach. We conducted a considerable histologic work to achieve good accuracy of the training dataset and to support the authenticity of RFC-based plaque characterization. Qualitative OCT plaque characterization also played an important role in determining the ROIs for training samples. From a translational perspective, the high resolving power and well-established plaque characterization methodology of OCT will be useful for procuring appropriate training samples in humans, which inevitably relies greatly on in vivo imaging data.

Elevated lipoprotein levels have been established as a major driver of atherosclerosis (40). However, despite the enhanced knowledge of atherosclerosis biology and intense lipid-lowering treatment, there remains a significant residual risk (41). Recent trials have provided convincing evidence that the interventions targeting residual inflammatory burden could further reduce the cardiovascular risk (42,43). Given the increasing recognition that plaque destabilization is a consequence of local imbalance between proinflammatory and proresolving immune pathways, novel therapeutics targeting inflammation resolution are currently being investigated (29,44,45). Our approach allowing simultaneous characterization of both lipids and MФs may offer valuable imaging insights into the complex interplay between lipids and atherogenic immune responses. This technique will be a promising imaging modality in the upcoming era of cardiovascular therapeutics targeting immune pathways on top of lipid-lowering therapy.

### Study limitations

This study is limited by the small number of animals included. Our findings based on swine models require validation to confirm whether human plaque components also have multispectrally distinguishable FL signatures. In this study, the component classification relied on 2 spectral data because the swine coronary artery exhibited weak fluorescence intensity in ch.3, similar to a previous study (16). Although we found significant differences in multiple or at least 1 FLIm parameter (ch.1 FL, ch.2 FL, or IR) across the 5 different components (Supplemental Table 2), substantial overlaps of the FL histograms in the absence of ch.3 data yielded a relatively low precision confusion matrix in the lipids+MФ-rich and MФ-rich regions. Given the previous autopsy study demonstrating strong ch.3 intensity and the role of the ch.3 FL in detecting plaque MФs (16,38), it is highly anticipated that the machine learning approach based on integrated information from all 3 spectral channels (ie, ch.1 FL, ch.2 FL, ch.3 FL, IR1 [ch.1/2], IR2 [ch.2/3], and IR3 [ch.3/1]) will enable a more sophisticated and accurate component characterization in humans. The thin-walled normal artery might be misclassified as fibrotic or lipidic, owing to the interference from the nearby myocardium or adipose tissue. Further validation and performance assessment of the current automated FLIm classification approach are needed for the detection of human atherosclerosis, which exhibits greater complexity and volume. Clinical studies are warranted to determine whether OCT-FLIm will provide value beyond standalone OCT or IVUS or near-infrared spectroscopy-IVUS for improving risk prediction.

## Conclusions

Coronary atherosclerosis is a highly complex, multifaceted disease process in which risk prediction has been challenging (4). Nevertheless, a recent progress in intravascular imaging has witnessed that the acquisition of additional biochemical information could allow identification of patients and arterial segments at risk for future coronary events (28). The present OCT-FLIm with machine learning–based analysis, allowing accurate characterization of both plaque morphology and biochemical components, is expected to open new avenues for high-risk plaque assessment.

Perspectives **COMPETENCY IN MEDICAL KNOWLEDGE:** Coronary plaque destabilization involves alterations in microstructure and biochemical composition; however, no imaging approach allows such comprehensive characterization. Herein, we demonstrated a simultaneous microstructural and biochemical assessment of high-risk plaques in coronary arteries in the beating heart using dual-modal OCT-FLIm. Furthermore, our machine learning–based FLIm approach enabled an automated, quantitative imaging of multiple key components relevant for plaque destabilization.

**TRANSLATIONAL OUTLOOK:** Further clinical studies are warranted to investigate whether this imaging strategy allows the identification of plaques and patients at risk for future coronary events.

## Funding Support and Author Disclosures

This work was supported by a grant from the Samsung Research Funding Center of Samsung Electronics (SRFC-IT1501-51 to Drs Yoo and J.W. Kim). Drs Oh, Yoo, and J.W. Kim own stock in Dotter, a company developing an OCT-FLIm intracoronary imaging system and catheter. All other authors have reported that they have no relationships relevant to the contents of this paper to disclose.

## Appendix

Supplemental Video 1 Real-time *in vivo* intracoronary dual-modal OCT-FLIm of a high-risk plaque.

Supplemental Data

Supplemental Video 2 Real-time *in vivo* OCT-FLIm of a normal artery.

Supplemental Video 3 Movie demonstrating the dedicated data analysis software. It provides real-time display of co-registered OCT-FLIm images and *en face* fluorescence lifetime maps of all three spectral channels.

Supplemental Video 4 RFC-applied OCT-FLIm of an atheromatous artery. It enables simultaneous comprehensive visualization of microstructural and biochemical properties of target plaques.

Supplemental Video 5 Three-dimensional flythrough-view movie of RFC-applied OCT-FLIm of an atheromatous artery

Supplemental Video 6 Movie of *in vivo* obtained from an atheromatous animal whose imaging data were not utilized for RFC training.
