# Supplementary material for: Comprehensive Assessment of High-Risk Plaques by Dual-Modal Imaging Catheter in Coronary Artery
Source: JACC Basic Transl Sci. 2021 Dec 27;6(12):948–60. doi: 10.1016/j.jacbts.2021.10.005 (PMC8733747; doi:10.1016/j.jacbts.2021.10.005)
Supplement: Supplemental Data [file mmc2.pdf]

SUPPLEMENTAL INFORMATION

Comprehensive assessment of high-risk plaque by dual-modal imaging catheter in coronary artery

Running title: Machine learning OCT-FLIm

Sunwon Kim[a,b,]†, Hyeong Soo Nam[c,] †, Min Woo Lee[d], Hyun Jung Kim[a],
Woo Jae Kang[c,e], Joon Woo Song[a], Jeongmoo Han[c], Dong Oh Kang[a], Wang-Yuhl Oh[c,e],
Hongki Yoo[c,]*, and Jin Won Kim[a,]*

[a]Multimodal Imaging and Theranostic Lab, Cardiovascular Center, Korea University Guro Hospital, Seoul, South Korea
[b]Department of Cardiology, Korea University Ansan Hospital, Ansan-si, South Korea
[c]Department of Mechanical Engineering, Korea Advanced Institute of Science and Technology, Daejeon, South Korea
[d]Depertment of Biomedical Engineering, Hanyang University, Seoul, South Korea
[e]KI for Health Science and Technology, Korea Advanced Institute of Science and Technology, Daejeon, South Korea

†contributed equally; *shared senior authorship

Address for Correspondence
Jin Won Kim, M.D., Ph.D.
Multimodal Imaging and Theranostic Lab, Cardiovascular Center, Korea University Guro Hospital, 148 Gurodong-ro, Guro-gu, Seoul 08308, South Korea
Tel: 82-2-2626-3021, Fax: 82-2-863-1109, E-mail: kjwmm@korea.ac.kr

Hongki Yoo, Ph.D.
Department of Mechanical Engineering, Korea Advanced Institute of Science and Technology, 291 Daehak-ro, Yuseong-gu, Daejeon 34141, South Korea
Tel: 82-42-350-3243, Fax: 82-42-350-8240, E-mail: h.yoo@kaist.ac.kr

Supplemental Methods and Results

High-speed multispectral FLIm-incorporated OCT imaging system

Our combined OCT-FLIm system generates combined images with information related to arterial morphology and multiple compositions in a high-speed manner (Supplemental Fig. 1).(1,2) In virtue of the high-speed and broadband wavelength-swept laser light source used in the OCT system, the high-speed and high-resolution OCT imaging was allowed by generating 120 k axial-lines per second with an axial resolution of 11 μm. In synchronous with OCT system, our FLIm collects spectrally- and temporally-resolved multispectral fluorescence emission excited by ultraviolet laser pulses with a wavelength of 355 nm, and measures the fluorescence lifetime of three different spectral channels with a precision under 40 picoseconds and an acquisition rate of 30 kHz. The dual-modal hybrid optical rotary joint comprised of custom-designed stationary and rotary collimators enables optical combination of broadband light including OCT NIR light (1250 ~ 1350 nm), FLIm excitation light (355 nm), and FLIm emission light (380 ~ 560 nm), and maintains a high optical efficiency across the broadband while rotating the rotor collimator at the speed of up to 100 rps.(2) The dual-modality OCT-FLIm catheter was fabricated by combining double-clad fiber (DCF) with ball lens probe attached at its distal end, torque coil, and transparent FEP sheath showing very low autofluorescence. Clinical translation of this dual-modality OCT-FLIm catheter can be effectively promoted due to its low catheter profile about 2.9 Fr, comparable to that of conventional OCT imaging catheter. The returning emission pulse was directed to the spectral resolving unit including a series of optical filters and dichroic mirrors, and spectrally and temporally separated for the multispectral fluorescence lifetime measurement. Electronically converted pulse sequences by a pre-amplifier-integrated photomultiplier module were recorded in synchronous with the corresponding OCT images.

The acquired synchronized OCT-FLIm information was processed to the combined image in real time and embedded to the storage for further post-processing by a dedicated multithreaded parallel programming.(1)

Animal models and experimental procedures

Yucatan minipigs (male, 3 months old, weighing 15 to 20 kg, n = 3; Optipharm, Korea) served as a model of coronary atherosclerosis (Supplemental Fig. 5). Accelerated coronary atherosclerosis was produced using a combination of streptozotocin (STZ)-induced diabetes, high-fat diets, and coronary balloon injury, as previously described.(3) After acclimation to atherogenic diet (2% cholesterol, 1.5% cholate, and 15% lard; Research Diet, Optipharm, Korea), experimental diabetes were induced by treating the pigs twice with low dose STZ (30mg/kg and 50 mg/kg of STZ with one week interval). The animals received dual anti-platelet treatment (aspirin 100mg and clopidogrel 75mg) prior to balloon injury experiment. After mild sedation via intramuscular administration of Azaperone (3-5mg/kg), Xylazine (1mg/kg), and Alfaxalone (3-5mg/kg), the animal was intubated and inhalation anesthesia (Isoflurane, 1.5-3%) was maintained. The carotid artery was exposed, and 6 F right Judkins right guiding catheter was introduced into left main coronary artery. Balloon overstretch injury (balloon to artery ratio: 1.3 - 1.5) was inflicted at the proximal to middle segment of the left anterior descending artery using semi-compliant coronary angioplasty balloons. The pig continued on atherogenic diet thereafter. ECG, pulse oximetry, and arterial pressure were continuously monitored during the experiment. After surgery, the animal received intramuscular non-steroidal anti-inflammatory drugs (Ketoprofen, 3mg/kg) and antibiotics (Enrofloxacin, 5mg/kg) for 3-5 days. The Institutional Animal Care and Use Committee of Korea University (KUIACUC-2017-0019 and KUIACUC-2018-0070) approved all animal procedures.

Immunohistochemistry to identify tissue component

Saline-perfused coronary arteries were dissected carefully preserving side branches, which served as a landmark when locating corresponding histology in longitudinal direction. The dissected arteries were then subjected to *ex vivo* OCT imaging to facilitate OCT-histology matching because histologic sections show closer resemblance to images obtained *ex vivo* from shrunken arteries than those obtained *in vivo*. Arteries were sectioned transversely at 3 mm intervals and each specimen was embedded in optimal cutting temperature compound (Tissue-Tek, Sakura Finetek, Japan). Frozen tissue segments were sectioned with a cryostat-microtome (Zeiss, German) at 10 µm thickness, yielding 12 consecutive sections as one set of slides for staining. Serially sectioned histological slides were indexed and arranged in order from proximal to distal. Unstained sets of slides were first examined microscopically for plaque morphology and immunostained serially if significant change in section morphology was identified. 10 µm-thickness frozen sections were incubated with macrophage-targeted monoclonal antibody (clone PM-2K, diluted 1:200, Abcam, Cambridge, UK) for the identification of macrophages.(4,5) A horseradish peroxidase-conjugated anti-mouse IgG (Envision + Kit-K4001; Dako Corp.) served as a secondary antibody, and diaminobenzidine substrate (Dako Corp.) was used for color development. Sister sections were stained using oil red O (ORO, ScyTek), and an anti-alpha smooth muscle actin polyclonal antibody (SMA, diluted 1:400, Abcam, Cambridge, UK) to label lipids and smooth muscle cells, respectively. Each section was scanned using by light microscopy (Axio Scan.Z1, Zeiss, Germany) and digitized. Digitized immunohistological sections were analyzed to quantify the relative contents (%) of macrophages, lipids, and, smooth muscle cells (SMC).

FLIm analyses according to different plaque types

A total of 826 OCT cross-sections obtained from 4 different pigs were reviewed. Obtained OCT frames were classified into normal-looking artery, fibrotic plaque, or high-risk plaque based on qualitative OCT characteristics by two imaging experts (SWK and JWS) who were blinded to FLIm data.(6,7) Fibrotic plaque was defined as an atheroma with >70% of plaque area exhibiting a homogenous, high-backscattering signal on OCT (Fig. 2). An atheroma with >70% of area having single or multiple ill-defined, signal-poor regions was considered a lipid-rich, i.e. high-risk plaque. Frames not otherwise classified by aforementioned criterion, or images with significant artefacts were excluded in the plaque-type-based FL analysis.

A total of 163 axial images from three different animals were thoroughly analyzed: high-risk plaque: 47 frames; fibrotic plaque: 43 frames; normal artery: 73 frames. The obtained ch.1 FL measurements from the OCT-based fibrotic plaques and normal arteries both yielded narrow single-peaked histograms with a similar mean FL and its frequency distribution ($p=0.062$, Supplemental Fig. 7). In contrast, those from the high-risk plaques yielded a wider histogram but significantly shorter mean ch.1 FL compared to normal controls (ch.1 FL: high-risk vs. fibrotic vs. normal: 4.22±0.14 vs. 4.46±0.05 vs. 4.42±0.05, ANOVA, $p<0.001$, Supplemental Fig. 7). In ch.2, while high-risk and fibrotic plaques yielded wide histograms with substantial overlaps, there were significant differences in both mean FL and its frequency distribution between three kinds of plaque type (ch.2 FL: high-risk vs. fibrotic vs. normal: 3.81±0.18 vs. 4.25±0.14 vs. 4.64±0.08, ANOVA, $p<0.001$). To clarify the FLIm readouts between three plaque types, intensity ratio (IR, fluorescence intensity ch.2/ch.1) was further compared. Collectively, the high-risk plaques were differentiable from the fibrotic plaques by ch.1, ch.2 FLs and IR values (IR: high-risk vs. fibrotic: 1.16±0.15 vs. 0.83±0.08, $p<0.001$). The fibrotic plaque was distinguishable from the normal artery by ch.2

FL and IR (both $p<0.001$). The high-risk plaque was also differentiable from the normal artery by ch.1, ch.2 FLs (both, $p<0.001$) and IR ($p=0.001$).

Random forest-based machine learning training, cross-validation, and performance evaluation

We introduced a Random forest classifier (RFC), which is a supervised machine learning classification method, for automatic characterization of plaque components (‘lipids+ MΦ’, ‘lipids’, ‘MΦ’, ‘fibrotic’, and ‘normal’) based on the obtained multispectral FLIm dataset (ch.1 and ch.2 FL, and IR). The RF classification algorithm is an ensemble supervised machine learning technique that combines a large number of weak decision tress classifiers and makes its final decision (classification result) by taking the majority voting of individual weak classifiers.[(8)] It also has advantages where it shows highly robust performance even on noisy data and less overfitting to its training dataset.(8) In this study, we empirically determined the number of tree classifier as 100 for the RF classification, which showed the least out-of-bag (OOB) error and the best performance. Based on the five-fold cross-validation, the classifier trained by the given dataset showed a generalized performance with the cross-validation error of 5.62% and OOB error of 5.59%. We also demonstrated its classification ability of each type against all other types via confusion matrix and multi-class receiver operation characteristics (ROC) curve, suggesting that the RFC could distinguish each plaque component with high accuracy, sensitivity, and specificity (Supplemental Table 3).

Supplemental Figures

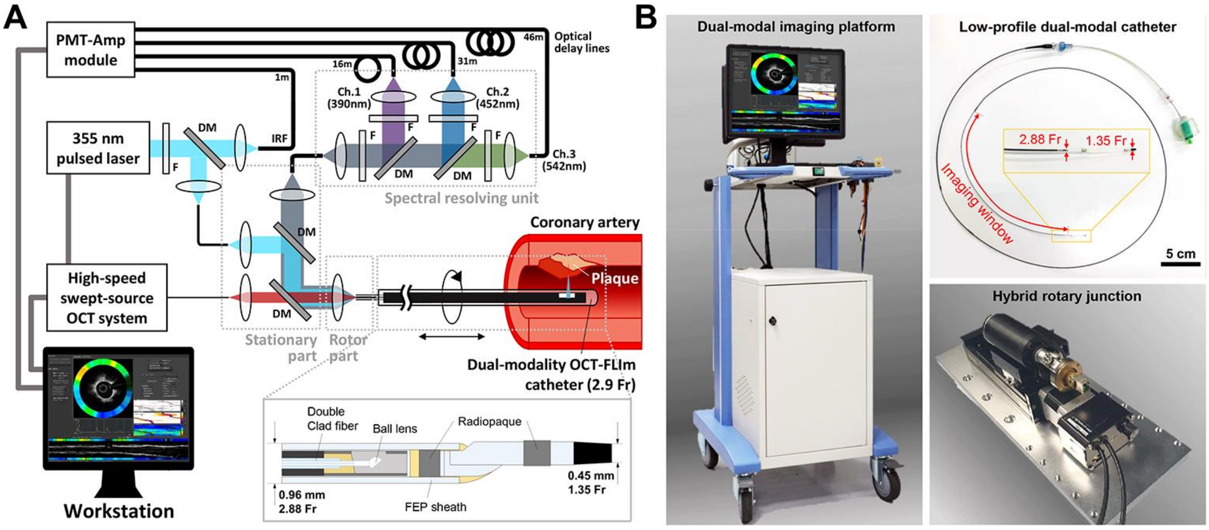

Supplemental Figure 1. Schematic diagram (A) and pictures (B) of the combined OCT-FLIm system and picture of 2.9 French low-profile dual-modal imaging catheter. A custom-built high-speed swept-source OCT system operates at a wavelength of 1,290 nm (a spectral bandwidth of 110 nm) with 120 kHz A-scan rate. The endogenous tissue autofluorescence is induced with an ultraviolet pulsed laser (355 nm, 1.64 nsec pulse width) every 4 A-scan acquisitions. The collected fluorescence emission is spectrally and temporally separated for the multispectral measurement of fluorescence lifetime in the spectral resolving unit. A double-clad fiber-based dual-modality OCT-FLIm catheter and rotary joint effectively combine both modalities. The outer diameter of the imaging catheter is about 2.9-French, which is comparable to that of clinically-available OCT catheter. PMT; photomultiplier tube, F; optical filter, DM; dichroic mirror, IRF; instrumental response function.

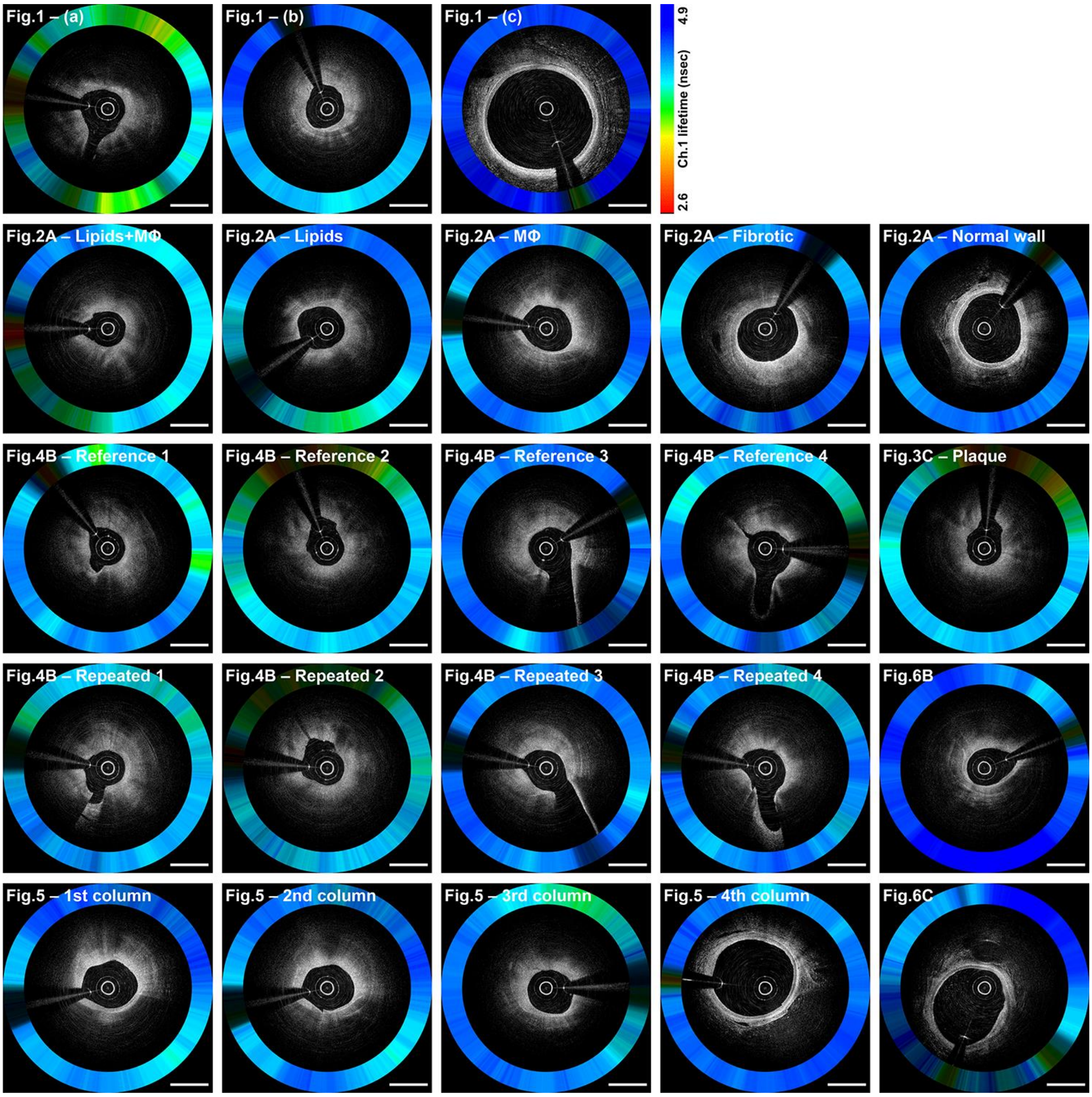

Supplemental Figure 2. OCT-FLIm cross-section images with ch.1 fluorescence lifetime used in Figs. 1 – 6 in the original manuscript. Scale bars, 1 mm.

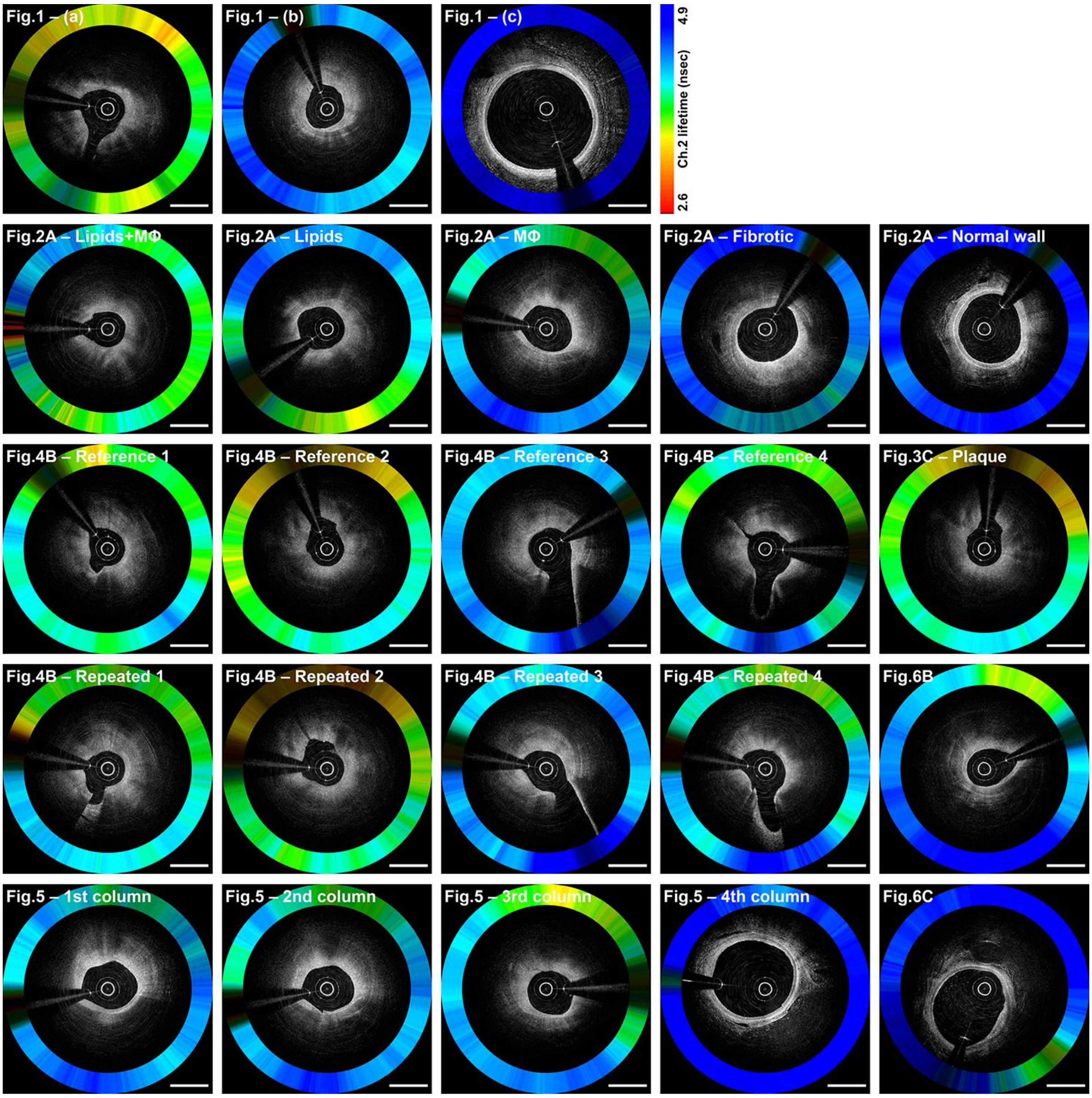

Supplemental Figure 3. OCT-FLIm cross-section images with ch.2 fluorescence lifetime used in Figs. 1 – 6 in the original manuscript. Scale bars, 1 mm.

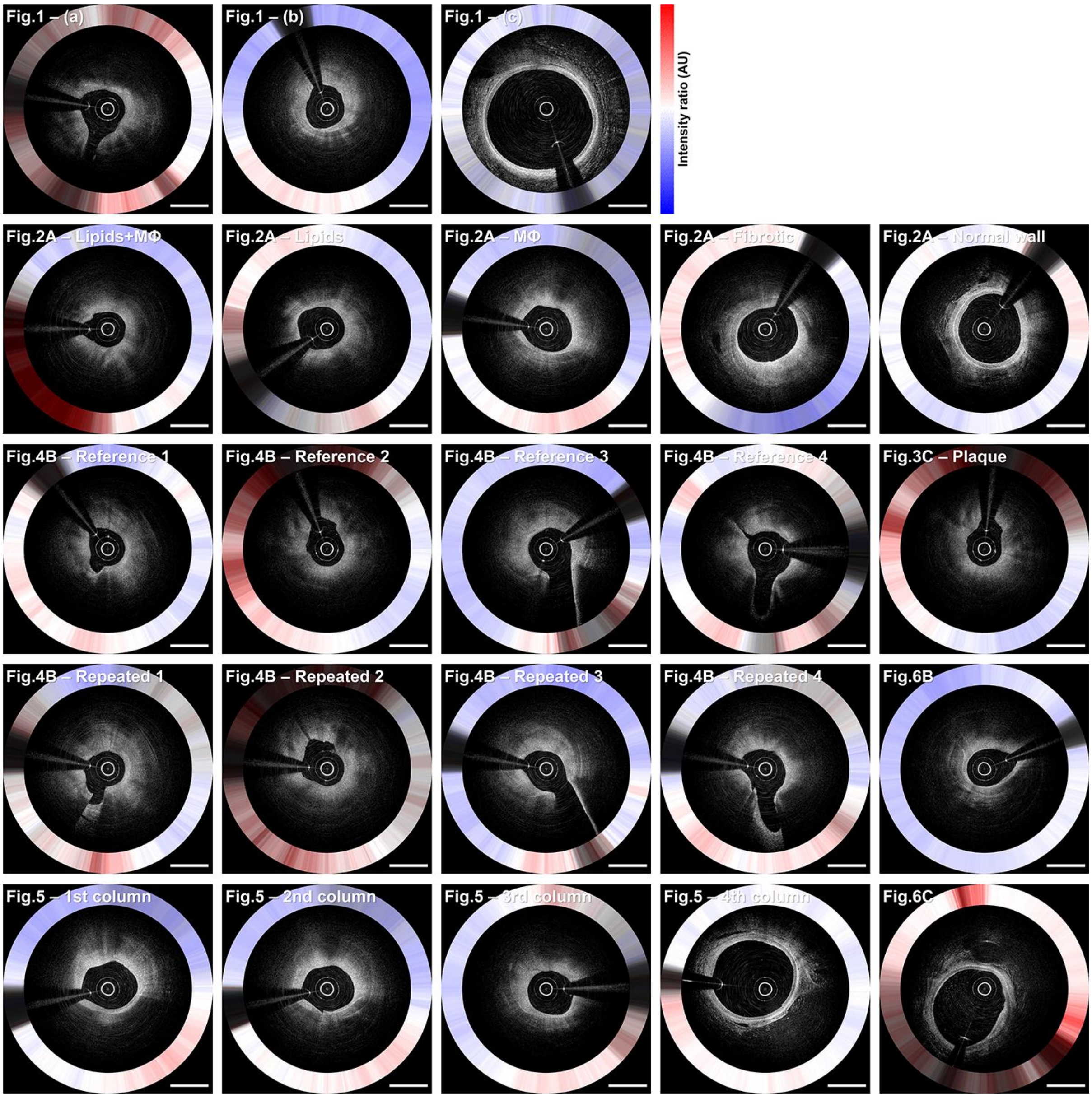

Supplemental Figure 4. OCT-FLIm cross-section images with intensity ratio (IR) used in Figs. 1 – 6 in the original manuscript. Scale bars, 1 mm.

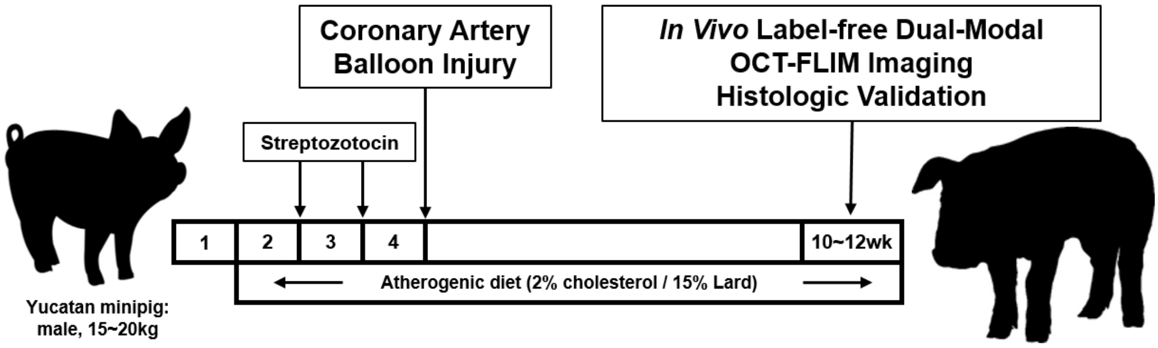

Supplemental Figure 5. Experimental scheme of a minipig model of accelerated coronary atherosclerosis.

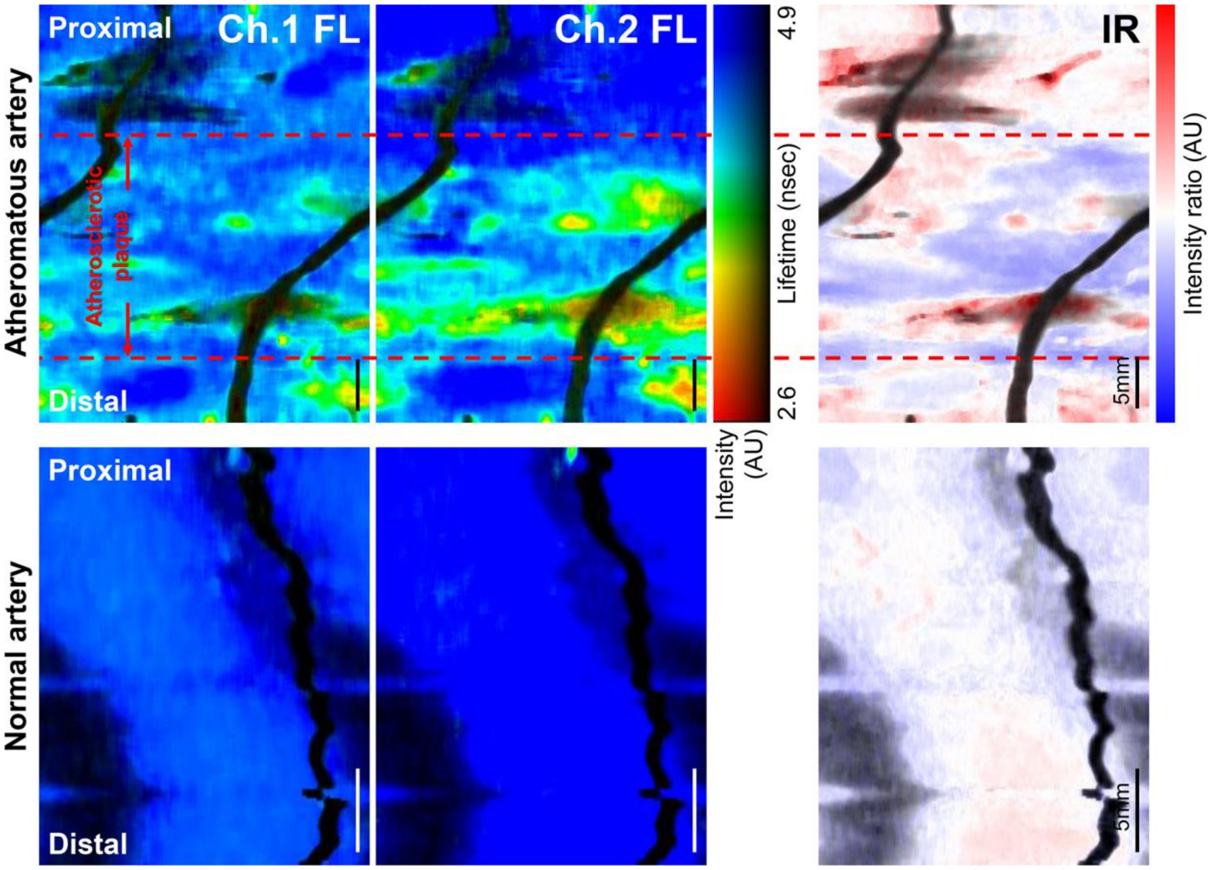

Supplemental Figure 6. *In vivo* FLIm *en face* maps. Each column indicates *en face* map of ch.1 FL, ch.2 FL, and intensity ratio (IR; ch.2 intensity/ch.1 intensity), respectively, and each row indicates data obtained from an atheroma and a normal artery, respectively. Scale bars, 5 mm.

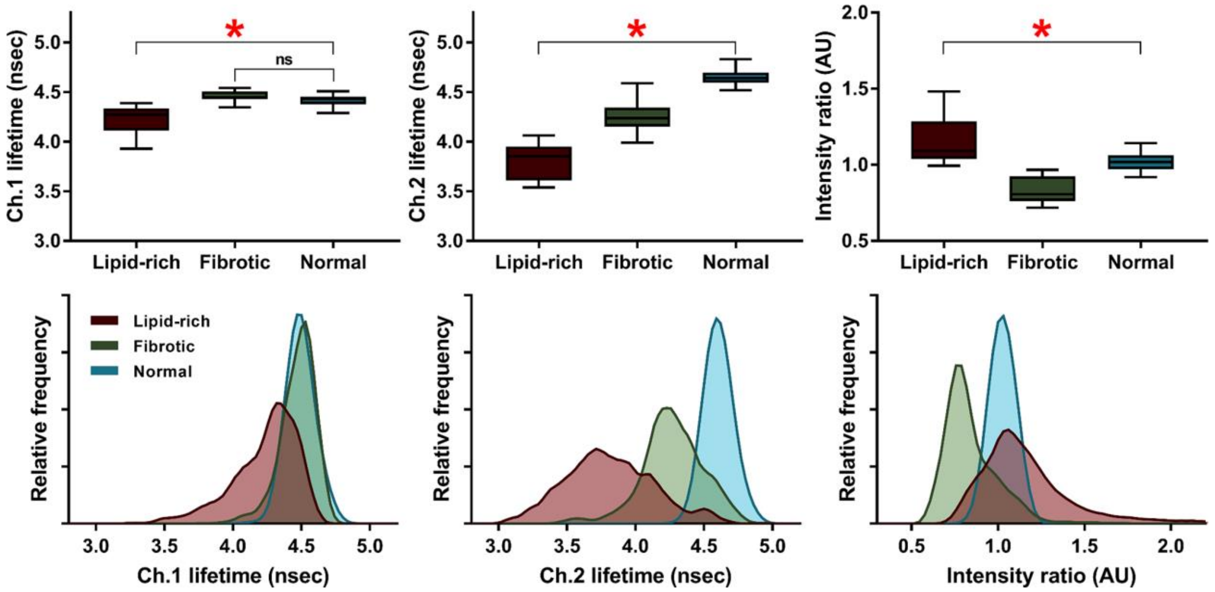

Supplemental Figure 7. Comparisons of multispectral FL measurements between the OCT-defined lipid-rich plaque vs. fibrotic plaque vs. normal artery. Boxplot center lines indicate medians; box edges represent the interquartile range; and whiskers extend to 1st or 3rd quartile plus 1.5 times interquartile range. Red asterisk denotes statistical significance (p<0.001) by ANOVA. The multiple comparison results were all p<0.001, unless specified or indicated as ns (non-significant).

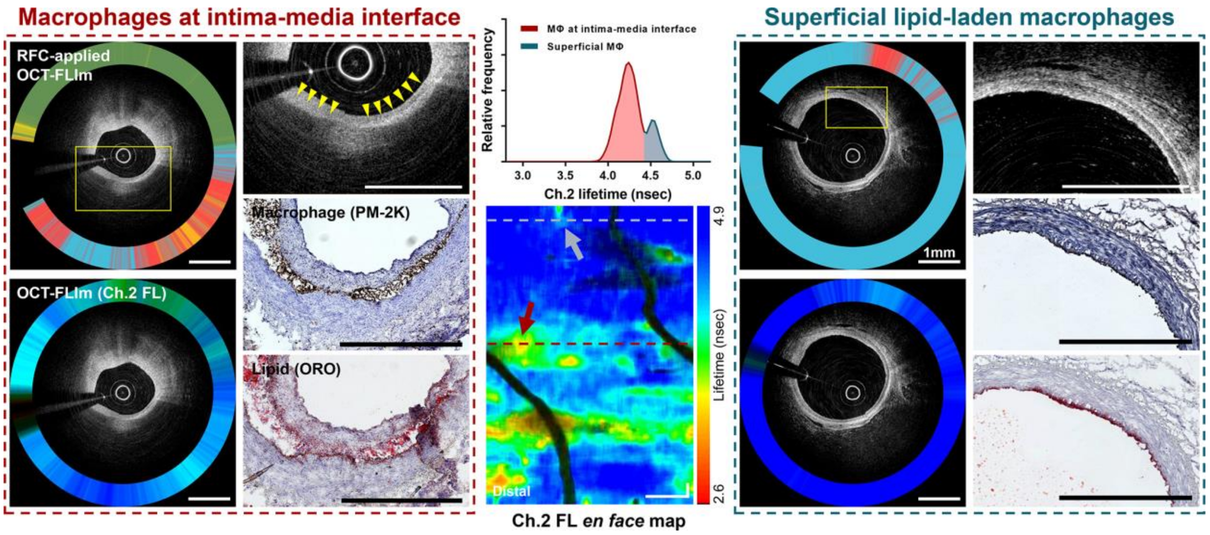

Supplemental Figure 8. Macrophages at intima-media interface vs. superficial lipid-laden macrophages. The ch.2 fluorescence lifetime measurements from the macrophages shows a double-peaked histogram, wherein one peak consisted of values from the lipid-laden macrophages clustered at the intima-media interface (left panel) and the other peak from those residing superficially at the vessel surface (right panel). Scale bars, 1 mm.

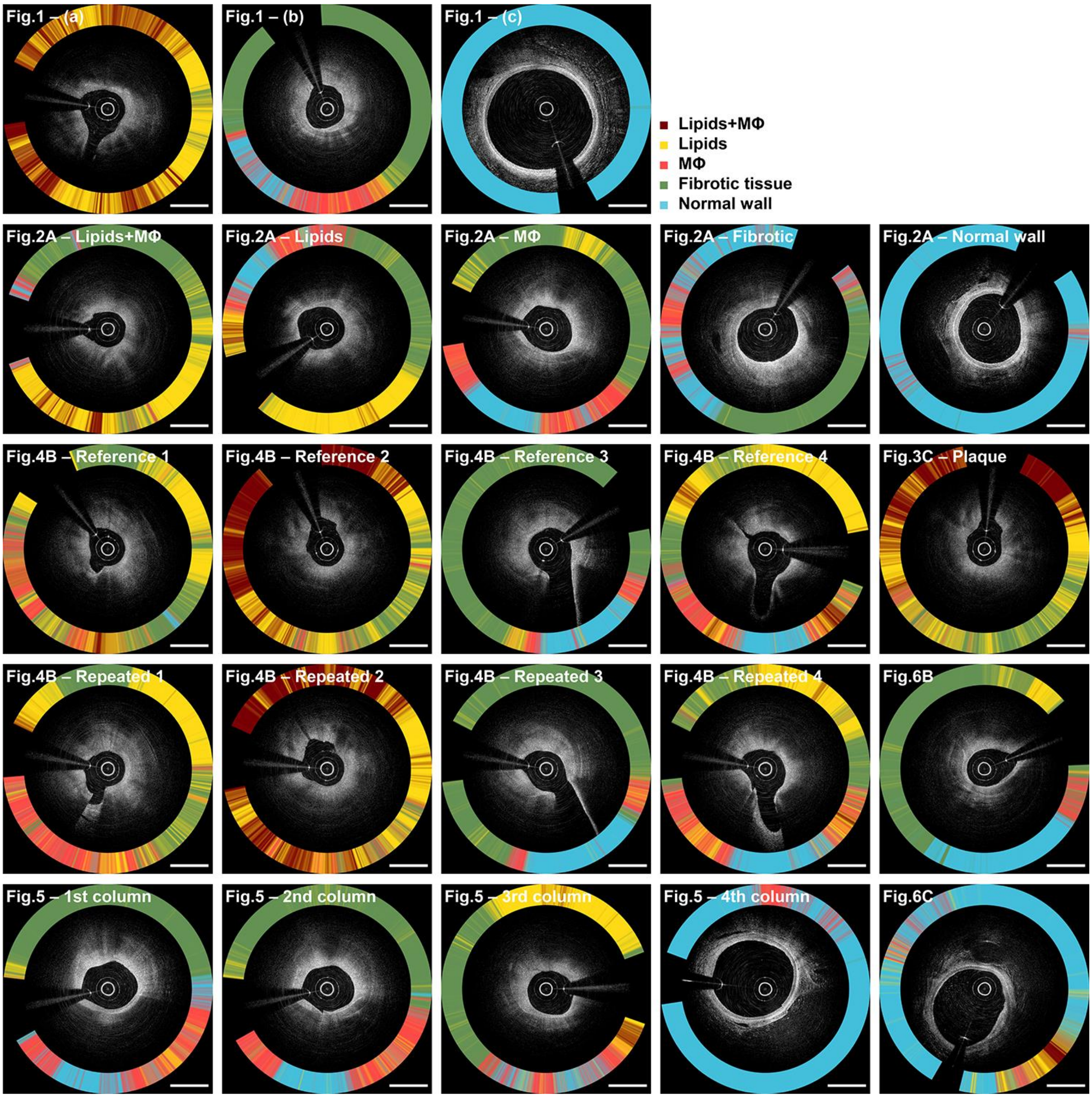

Supplemental Figure 9. Cross-section images with RFC-based classification results. Scale bars, 1 mm.

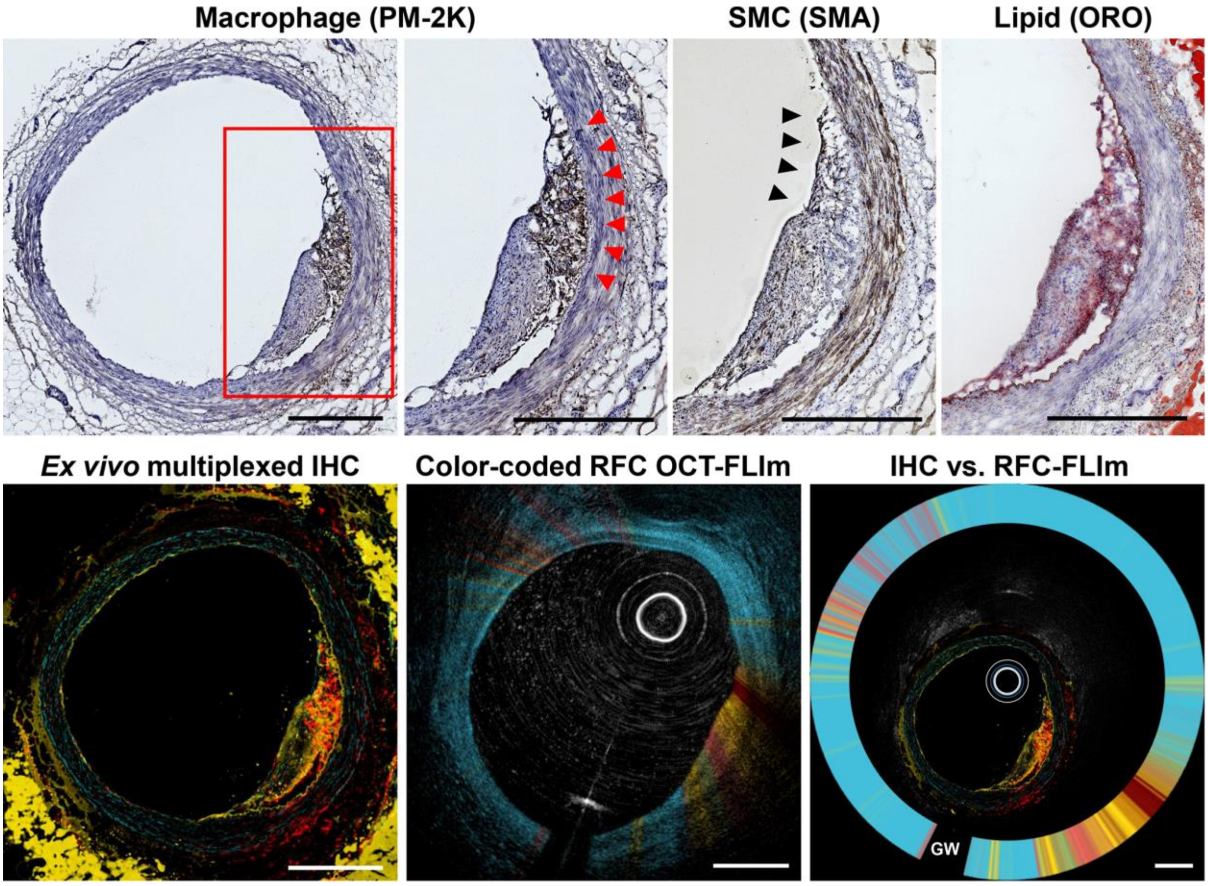

Supplemental Figure 10. Immunohistopathological validation on a focal plaque. Focal plaque showing extensive infiltration of lipid-laden macrophages and thinning of overlying smooth muscle cell layer at the plaque shoulder. Scale bars, 500 μm.

Supplemental Tables

Supplemental Table 1. Measured mean values of multispectral FLIm parameters (ch.1 and ch.2 lifetimes, and intensity ratio) across five different plaque components. Values are expressed as mean ± standard deviation.

	Lipids+MΦ	Lipid-rich	MΦ-rich	Fibrotic	Normal
Ch.1 lifetime (nsec)	3.786±0.227	3.906±0.195	4.268±0.081	4.399±0.088	4.422±0.048
Ch.2 lifetime (nsec)	3.451±0.172	3.638±0.182	4.263±0.142	4.224±0.143	4.644±0.086
Intensity ratio (AU)	1.350±0.129	1.092±0.106	1.086±0.076	0.742±0.082	1.022±0.059

Supplemental Table 2. Results of multiple comparison of multispectral FLIm parameters (ch.1 and ch.2 lifetimes, and intensity ratio) across five different plaque components.

Ch.1 lifetime	Lipids+MΦ	Lipid-rich	MΦ-rich	Fibrotic	Normal
Lipids+MΦ		> 0.99	< 0.001	< 0.001	< 0.001
Lipid-rich			0.001	< 0.001	< 0.001
MΦ-rich				< 0.001	< 0.001
Fibrotic					> 0.99
normal					

Ch.2 lifetime	Lipids+MΦ	Lipid-rich	MΦ-rich	Fibrotic	Normal
Lipids+MΦ		0.72	< 0.001	< 0.001	< 0.001
Lipid-rich			< 0.001	< 0.001	< 0.001
MΦ-rich				> 0.99	< 0.001
Fibrotic					< 0.001
Normal					

Intensity ratio	Lipids+MΦ	Lipid-rich	MΦ-rich	Fibrotic	Normal
Lipids+MΦ		< 0.001	< 0.001	< 0.001	< 0.001
Lipid-rich			> 0.99	< 0.001	0.022
MΦ-rich				< 0.001	0.14
Fibrotic					< 0.001
Normal					

Supplemental Table 3. Measured area under the curve (AUC), sensitivity, and specificity for plaque components classification using the random forest classifier.

	Lipids+MΦ	Lipid-rich	MΦ-rich	Fibrotic	Normal
AUC	0.9886	0.9887	0.9904	0.9981	0.9991
Sensitivity	0.9838	0.9704	0.9742	0.9914	0.9910
Specificity	0.9486	0.9282	0.9534	0.9714	0.9842

Supplemental References

1. Nam HS, Kang WJ, Lee MW et al. Multispectral analog-mean-delay fluorescence lifetime imaging combined with optical coherence tomography. Biomed Opt Express 2018;9:1930-1947.
2. Lee MW, Song JW, Kang WJ et al. Comprehensive intravascular imaging of atherosclerotic plaque in vivo using optical coherence tomography and fluorescence lifetime imaging. Sci Rep 2018;8:14561.
3. Kim S, Lee MW, Kim TS et al. Intracoronary dual-modal optical coherence tomography-near-infrared fluorescence structural-molecular imaging with a clinical dose of indocyanine green for the assessment of high-risk plaques and stent-associated inflammation in a beating coronary artery. European heart journal 2016;37:2833-2844.
4. Zeng L, Takeya M, Ling X, Nagasaki A, Takahashi K. Interspecies reactivities of anti-human macrophage monoclonal antibodies to various animal species. The journal of histochemistry and cytochemistry : official journal of the Histochemistry Society 1996;44:845-53.
5. Takeya M, Tsuchiya T, Shimokawa Y, Takahashi K. A new monoclonal antibody, PM-2K, specifically recognizes tissue macrophages but not blood monocytes. The Journal of pathology 1991;163:315-21.
6. Tearney GJ, Regar E, Akasaka T et al. Consensus standards for acquisition, measurement, and reporting of intravascular optical coherence tomography studies: a report from the International Working Group for Intravascular Optical Coherence Tomography Standardization and Validation. J Am Coll Cardiol 2012;59:1058-72.
7. Otsuka F, Joner M, Prati F, Virmani R, Narula J. Clinical classification of plaque morphology in coronary disease. Nat Rev Cardiol 2014;11:379-89.
8. Breiman L. Random forests. Mach Learn 2001;45:5-32.
